# A Chimeric Humanized Mouse Model by Engrafting the Human Induced Pluripotent Stem Cell-Derived Hepatocyte-Like Cell for the Chronic Hepatitis B Virus Infection

**DOI:** 10.3389/fmicb.2018.00908

**Published:** 2018-05-08

**Authors:** Lunzhi Yuan, Xuan Liu, Liang Zhang, Xiaoling Li, Yali Zhang, Kun Wu, Yao Chen, Jiali Cao, Wangheng Hou, Jun Zhang, Hua Zhu, Quan Yuan, Qiyi Tang, Tong Cheng, Ningshao Xia

**Affiliations:** ^1^State Key Laboratory of Molecular Vaccinology and Molecular Diagnostics, School of Public Health, Xiamen University, Xiamen, China; ^2^National Institute of Diagnostics and Vaccine Development in Infectious Diseases, School of Public Health, Xiamen University, Xiamen, China; ^3^School of Life Sciences, Xiamen University, Xiamen, China; ^4^Department of Microbiology and Molecular Genetics, New Jersey Medical School, Rutgers University, Newark, NJ, United States; ^5^Department of Microbiology, Howard University College of Medicine, Washington, DC, United States

**Keywords:** stem cells, hepatocyte-like cells, liver humanized mouse, hepatitis B virus, infectious animal model, hHLC-FRGS

## Abstract

Humanized mouse model generated by grafting primary human hepatocytes (PHHs) to immunodeficient mouse has contributed invaluably to understanding the pathogenesis of hepatitis B virus (HBV). However, the source of PHHs is limited, which necessitates the search for alternatives. Recently, hepatocyte-like cells (HLCs) generated from human induced pluripotent stem cells (hiPSCs) have been used for *in vitro* HBV infection. Herein, we developed a robust human liver chimeric animal model to study *in vivo* HBV infection by engrafting the hiPSC-HLCs to *Fah^-/-^Rag2^-/-^IL-2Rγc^-/-^* *SCID* (FRGS) mice. After being optimized by a small molecule, XMU-MP-1, the hiPSC-HLCs engrafted FRGS (hHLC-FRGS) mice displayed approximately 40% liver chimerism at week 6 after engraftment and maintained at this level for at least 14 weeks. Viremia and HBV infection markers include antigens, RNA, DNA, and covalently closed circular DNA were detectable in HBV infected hHLC-FRGS mice. Furthermore, hiPSC-HLCs and hHLC-FRGS mice were successfully used to evaluate different antivirals. Therefore, we established a humanized mouse model for not only investigating HBV pathogenesis but also testing the effects of the anti-HBV drugs.

**Highlights**:

(1) The implanted hiPSC-HLCs established a long-term chimerism in FRGS mice liver.

(2) hHLC-FRGS mice are adequate to support chronic HBV infection with a full viral life cycle.

(3) hiPSC-HLCs and hHLC-FRGS mice are useful tools for evaluation of antivirals against HBV infection *in vitro* and *in vivo*.

**Research in Context**

To overcome the disadvantages of using primary human hepatocytes, we induced human pluripotent stem cells to hepatocyte-like cells (hiPSC-HLCs) that developed the capability to express important liver functional markers and critical host factors for HBV infection. The hiPSC-HLCs were permissive for the HBV infection and supported a full HBV replication. The hiPSC-HLCs were then engrafted to immunodeficient mouse to establish a chimeric liver mouse model, which was capable of supporting HBV infection *in vivo* and evaluating the effects of antiviral drugs. Our results shed light into improving the cellular and animal models for studying HBV and other hepatotropic viruses.

## Introduction

Hepatitis B virus (HBV) is a globally spreading pathogen, infects more than 350 million people, and relates to liver cirrhosis and hepatocellular carcinoma (HCC). A productive HBV infection is not only species-dependent but also hepatocyte-specific (Seeger and Mason, 2000). Because of its narrow host range and limited cell or tissue tropism, HBV fails to infect small animals; hence, most information was achieved from non-human primates or from clinical studies (Gripon et al., 1988; Galle et al., 1994; Kock et al., 2001). The first line cells used for HBV infection has been the primary human hepatocytes (PHHs). After PHHs were engrafted in the liver of an immune-deficient mouse, a chimeric mouse model has been generated for *in vivo* experiment to study the HBV pathogenesis (de Jong et al., 2010; Dandri and Petersen, 2012; Allweiss and Dandri, 2016; Thomas and Liang, 2016). However, the drawbacks of using PHHs include the limited source of their availability, donor variability, inability to expand *in vitro*, rapid de-differentiation, and poor susceptibility to a long-term HBV infection (Gripon et al., 1988). An alternative cell line, the bipotential liver progenitor cell (HepaRG) was induced for 1 month to achieve the hepatic differentiation. The differentiated HepaRG cells with a mixed cell phenotypes were used for HBV infection and showed a low HBV infection efficiency, which limited its use for HBV study (Gripon et al., 2002; Hantz et al., 2009). Other trials, such as receptor overexpressing hepatoma cells, were not optimal due to their transformed nature, deficiency of innate immunity, and low expression of important hepatic makers (e.g., hALB) and critical host factors required for HBV infection [e.g., human retinoic X receptor (hRXR) and hepatocyte nuclear factor 4 alpha (hHNF4α); Ni and Urban, 2017].

Human stem cell-derived hepatocyte-like cells (hHLCs) have been demonstrated to be a promising novel tool for studying HBV and other hepatotropic virus infections *in vitro* (Paganelli et al., 2013; Carpentier et al., 2014; Shlomai et al., 2014; Helsen et al., 2016; Ni and Urban, 2017; Xia et al., 2017; Yan et al., 2017). These hHLCs are physiologically similar to PHHs, have a slow de-differentiation, are available for a long-term *in vitro* infection, and can be genetically manipulated (Ni and Urban, 2017). Previous studies have demonstrated that hHLCs derived from human stem cells were expandable in the mouse liver and were permissive for the hepatitis C virus (HCV) infection (Basma et al., 2009; Carpentier et al., 2014; Yan et al., 2017). We were curious whether the human induced pluripotent stem cells to hepatocyte-like cell (hiPSC-HLC) engrafted mouse is capable of supporting the HBV infection. To that end, we generated hiPSC-HLCs as previously described and engrafted hHLCs to immunodeficient *Fah^-/-^Rag2^-/-^IL-2Rγc^-/-^* *SCID* (FRGS) mice to generate a human liver chimeric mouse model: hiPSC-HLCs engrafted FRGS (hHLC-FRGS). The functional small molecule FH1 was used to enhance hepatic maturation and maintain the hepatic differentiation state of hiPSC-HLCs (Shan et al., 2013). The functional small molecule XMU-MP-1 was used to augment the expansion of implanted hiPSC-HLCs in the mouse liver (Fan et al., 2016). Furthermore, hHLC-FRGS mice were challenged with HBV and used to evaluate different types of antivirals, including HBV entry inhibitor Myrcludex B (Volz et al., 2013; Blank et al., 2016) and the clinical nucleoside analog entecavir (ETV). Overall, hiPSC-HLCs and hHLC-FRGS mice provided a novel infectious cell culture system and novel animal model, respectively, to study the HBV replication, pathogenesis, and antiviral therapy.

## Materials and Methods

### Hepatic Differentiation of hiPSCs

The human induced pluripotent stem cells (hiPSCs named GZF induced from human fibroblasts) were obtained from the Key Laboratory of Regenerative Biology, Chinese Academy of Sciences, and cultured as previously described (Esteban et al., 2010). The hepatic differentiation of hiPSCs was performed following a three-step protocol adapted from previous studies (Basma et al., 2009; Carpentier et al., 2014, 2016; Xia et al., 2017). First, hiPSCs at a confluence of 60–70% were induced to definitive endoderm by STEMdiff^TM^ Definitive Endoderm Kit (StemCell Technologies) within 4 days according to the manufacturer instructions. Briefly, monolayer hiPSCs were suspended after treatment of Accutase (StemCell Technologies, cat # AT104) and seeded onto a six-well plate with two million cells per well in the definitive endoderm basal medium. The plate was pre-coated with Matrigel (0.125 mg/mL, BD Biosciences, cat # 356234) and the definitive endoderm basal medium contained supplements A and B (STEMdiff^TM^ Definitive Endoderm Kit, StemCell Technologies, cat # 05110) for the first day and then only B for three additional days (with a daily medium change). For hepatocyte formation, definitive endoderm cells were suspended after treated with Accutase and seeded back to six-well plates (pre-coated with Matrigel) in the hepatic differentiation medium: high glucose Dulbecco’s modified Eagle’s medium (DMEM, Sigma-Aldrich) for 3 days. From day 4 to 10, the functional components [1% dimethyl sulfoxide (DMSO), 10^-7^ M dexamethasone (DEX, LONZA, cat # CC4182-1), 100 ng/mL hepatocyte growth factor (HGF, Sigma-Aldrich, cat # 1404), 5 μg/mL of small molecule FH1 from APExBIO (cat # BRD-K4477), 5 μg/mL insulin (LONZA, cat # CC-4321BB), 500 μM basic fibroblast growth factor (bFGF, LONZA, cat # CC4182-3), epidermal growth factor (EGF) from HCM BulletKit of LONZA (cat # CC4317BB)] were added to the medium. To achieve a mature hepatic differentiation, the hepatoblast-like cells were briefly treated with 2 mg/mL collagenase (Sigma-Aldrich, cat # A004186-0001) as previously described (Shi et al., 2016) and cultured in hepatic maturation medium for an additional 4 days (day 11–14). The hepatic maturation medium was Williams’ Medium E (WME, GIBCO, cat # A1217601) with 10% fetal bovine serum (FBS, GIBCO), 500 μM oncostatin-M (OSM, LONZA, cat # CC4182-4), 500 μM R3-IGF-1 (LONZA, cat # CC4182-5), 500 μM ascorbic acid (LONZA, cat # CC-4301BB), 500 μM hydrocortisone (HC, LONZA, cat # CC-4335BB) from HCM BulletKit of LONZA, and the functional components in hepatic differentiation medium. For the hepatic specification and maturation, medium was changed every 2 days for 2 weeks.

### *In Vitro* Culture of hiPSC-HLCs

To maintain the hiPSC-HLCs in the differentiated hepatic state, hiPSC-HLCs were cultured in WME with 10% FBS, 1% DMSO, 10^-7^ M DEX, 5 × 10^-5^ M HC, 5 μg/mL insulin, and 5 μg/mL FH1.

### Ethics Statement

All animal experiments were carried out in strict compliance with the Animal Welfare Act, PHS Policy, and the standards of the American Association for the Accreditation of Laboratory Animal Care and other national statutes and regulations relating to animals. The animal using protocol was approved by the Institutional Animal Care and Use Committee (IACUC) and Laboratory Animal Management Ethics Committee at Xiamen University (Protocol Number: XMULAC20160049).

### Animal Study

To obtain the FRGS mice, *Fah^-/-^Rag2^-/-^IL-2Rγc^-/-^* (FRG) mice described in our previous studies (Fan et al., 2016; Zhang et al., 2016) were bred with *BALB/c SCID* mice (Shanghai SLAC Laboratory Animal Co., Ltd., China) in specific pathogen free (SPF) laboratory in Animal Centre of Xiamen University.

### Generation of hHLC-FRGS Mice

FRGS mice were anesthetized with isoflurane and received splenic injection of 3 × 10^6^ hiPSC-HLCs as previously described (Azuma et al., 2007; Bissig et al., 2010). Liver injury was conducted by 2-(2-nitro-4-trifluoromethylbenzoyl)-1,3-cyclohexanedione (NTBC, SOBI, Sweden, cat # 66607-1005-6) cycling (Azuma et al., 2007) and anti-mouse CD95 antibody JO2 (BD Biosciences) (Bility et al., 2014) to kill part of mouse liver cells so as to provide space for the implanted hiPSC-HLCs. NTBC was gradually reduced in drinking water from week 7 to 14 and briefly provided at days 7, 21, and 35 post-engraftments. FRGS was injected intraperitoneally with 0.2 mg/kg JO2 at -1, 7, 14, 21, 28, and 35 days post-engraftment. To enhance the expansion of the implanted hiPSC-HLCs, mice were treated with XMU-MP-1 (1 mg/kg, dissolved in 0.1% citric acid aqueous solution containing 20% Kolliphor HS 15, APExBIO, cat # A8735) intraperitoneally together with the JO2 (BD Biosciences, cat # 554255) for six times from week 0 to 6 post-engraftment. To maintain a relatively stable liver chimerism, hHLC-FRGS mice received 100% NTBC in drinking water without other treatments from week 6 to 20 post-engraftment.

### HBV Infection

Genotype A, B, C, or D of HBV (isolated and purified by our laboratory previously) was propagated and amplified in HBV-replicating stable cell lines (HepG2-HBV1.3-A, -B, -C, or -D) generated from HepG2 cell (ATCC, cat # HB-8065) in our previous studies (Wu et al., 2016) and purified as previously described (Gripon et al., 2005). For the *in vitro* studies, hiPSC-HLCs were incubated with each genotype of HBV at a multiplicity of infection (MOI) of 200 (genome equivalents) in the cell culture medium supplemented with 4% PEG 8000 for 20 h at 37°C. Then, the cells were washed three times with the cell culture medium and the medium was changed every 2 days. For the *in vivo* HBV infection, hHLC-FRGS mice that have 2000 μg hALB per mL serum or above were inoculated intraperitoneally with 1 × 10^6^ DNA copies of infectious HBV dissolved in normal saline. During the time of HBV infection, hHLC-FRGS mice received 100% NTBC in drinking water, and samples were collected at indicated time points.

### Antiviral Experiments

To examine the anti-HBV effects of drugs *in vitro*, the HBV-infected hiPSC-HLCs were seeded in 24-well plates and treated with 100 nM Myrcludex B (GL Biochem, Shanghai, cat # GLS170707-QRR) synthesized as previously described (Volz et al., 2013), 0.5 μM ETV (Sigma-Aldrich, cat # 1235966), 2 μM host-targeting agents PA452 (TOCRIS, cat # 5086) and 10 μM genistin (APExBIO, cat # N1860), and B245 siRNA plasmid target HBV genome described in our previous studies (Zhang et al., 2010). To test the effects of antiviral spreading *in vivo*, hHLC-FRGS mice were injected weekly subcutaneously (s.c.) with Myrcludex B (2 mg/g) with or without ETV in drinking water as previously described from week 1 to 12 post-infection of HBV (Volz et al., 2013; Zhang et al., 2016).

### HBV Total DNA and cccDNA Examination by Southern Blot Assay

First, viral DNA was isolated from the HBV-infected cell culture, or the HBV-infected mice livers at 12 weeks post-infection (w.p.i.), or the cells and livers of the uninfected controls by two methods: the “core DNA” method for HBV total DNA including the relaxed circular (RC) and double stranded (DS) DNA and the optimized “Hirt” method (Ren and Nassal, 2001) for the covalently closed circular DNA (cccDNA).

The “core DNA” method was to isolate and purify the HBV DNA from intracellular core particles according to the protocol described previously (Ling and Harrison, 1997) with minor modifications. Briefly, the cells or the homogenized liver tissues were lysed in 300 μL isotonic lysis buffer (10 mmol/L Tris–HCl, pH 7.5, 1 mmol/L EDTA, 150 mmol/L NaCl, 0.5% Nonidet P-40) per 60-mm dish. The nuclei were removed from the lysates by centrifugation for 10 min at 14,000 rpm. The cytoplasmic HBV DNA was purified by digestion of proteins with 200 g/mL of proteinase K in the presence of 0.5% of sodium dodecyl sulfate (SDS) at 37°C overnight, phenol–chloroform extraction, and ethanol precipitation. One-third of the purified DNA was fractionated in a 1% agarose gel, transferred to a nylon membrane, and hybridized with a DIG-labeled DNA probe that covered the entire HBsAg gene.

An optimized “Hirt” method was used to isolate cccDNA. The cells or the homogenized liver tissues were resuspended in lysis buffer containing 0.2% NP-40, mixed with an equal volume of 0.15 M NaOH containing 6% SDS, incubated at 37°C for 20 min, and neutralized by adding 3 M K^+^ acetate (pH 5.5) to a final concentration of 0.6 M. After 30 min on ice, cellular debris and chromosomal DNA were removed by centrifugation at 20,000 *g* for 15 min at 4°C. After phenol extraction, soluble nucleic acids in the supernatant were precipitated by adding 0.7 volumes of isopropanol. The pellets were resuspended in 500 μL of restriction buffer 4 (New England Biolabs) containing 40 U of *Hpa*I/mL, 400 g of RNase A/mL, and 300 U of Plasmid-Safe DNase (Epicenter Technologies)/mL and incubated at 37°C for 4–6 h. Subsequently, a second NaOH treatment was performed by adding 0.2 M NaOH to 0.05 N final concentration. After 10 min at 37°C, the reaction was neutralized by adding 3 M K^+^ acetate (pH 5.5) to 0.6 M final concentration, the samples were extracted with phenol, and nucleic acids were ethanol precipitated. Southern blot analysis was performed as described above.

Quantification of HBV RNA and cccDNA were performed as previously described (Singh et al., 2004; Wang et al., 2016).

### Flow Cytometry (FACS) Assay

The cells were fixed with 4% paraformaldehyde and permeabilized by 0.1% Triton X-100. The cells were then incubated with indicated antibodies at 4°C for 30 min, washed with PBS twice, and analyzed with a fluorescence activated cell sorting (FACS) instrument (Facsaria III, BD Biosciences). The antibodies were listed in Supplementary Table 1.

### Statistical Analysis

Student’s unpaired two-tailed *t*-tests were performed with GraphPad Prism 7.0 (GraphPad Software). Data are presented as the means ± SEM. Two-sided *P*-values < 0.05 were considered significant: ^∗^*P* < 0.05, ^∗∗^*P* < 0.01, ^∗∗∗^*P* < 0.001, ^∗∗∗∗^*P* < 0.0001.

More details of reagents, antibodies, primers, probes, sample measurement, and analysis are provided in section “Supplementary Materials and Methods” in Supplementary Material.

## Results

### Generation and Characterization of hiPSC-HLCs

**FIGURE 1:**
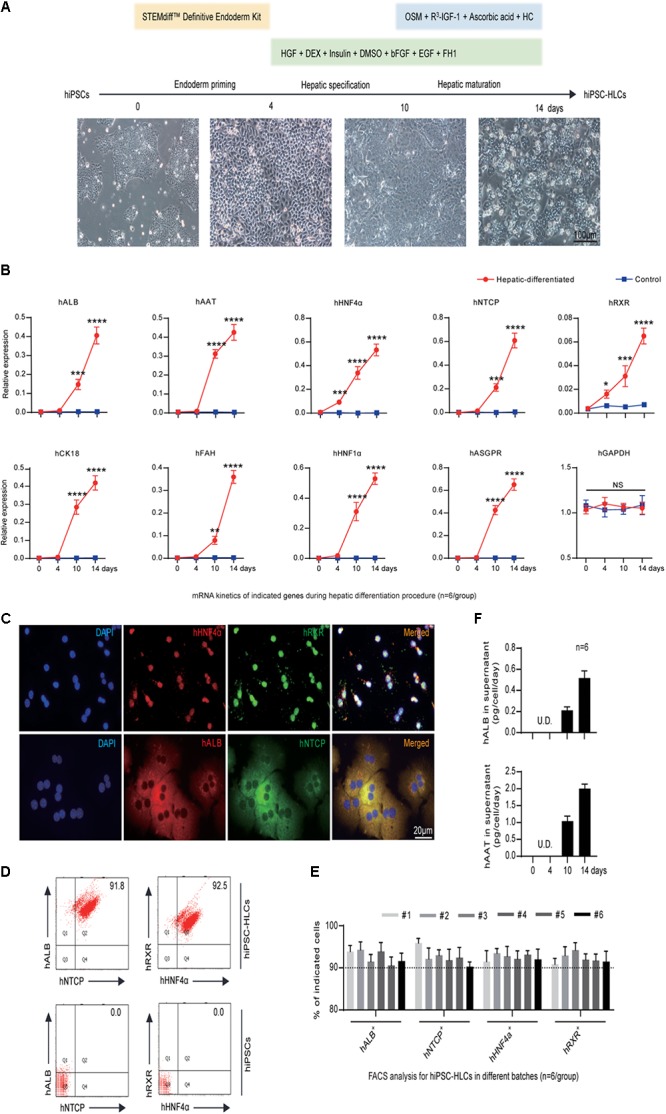
Generation and characterization of hiPSC-HLCs. **(A)** The three-step hepatic differentiation includes endoderm priming (days 0–3), hepatic specification (days 4–10), and maturation (days 11–14). Cell morphology at days 0, 4, 10, and 14 is also shown (bar = 100 μm). **(B)** Fold changes of human hepatic specific gene transcriptions during the hepatic differentiation by qRT-PCR (*n* = 6). The hGAPDH gene served as the control. The primers were as shown in Supplementary Table 2. **(C)** Immunocytochemistry (ICC) to visualize the hepatic specific proteins hALB, hNTCP, hRXR, and hHNF4α in differentiated hiPSC-HLCs (bar = 20 μm). **(D)** Representative FACS dot plots for positive ratios of hALB, hNTCP, hRXR, and hHNF4α in mature hiPSC-HLCs and in hiPSC (as negative control). **(E)** Statistics for different batches of hiPSC-HLCs (*n* = 6). **(F)** The levels of hALB and hAAT in culture supernatant of differentiated hiPSC-HLCs (*n* = 6). ^∗^*P* < 0.05, ^∗∗^*P* < 0.01, ^∗∗∗^*P* < 0.001, ^∗∗∗∗^*P* < 0.0001.

Human pluripotent stem cells to hepatocyte-like cells were generated from hiPSCs by a three-step procedure adapted from previous studies: endoderm priming, hepatic specification, and maturation within 14 days (Basma et al., 2009; Carpentier et al., 2014, 2016; Xia et al., 2017) as shown in **Figure 1A**. To enhance the hepatic properties of hiPSC-HLCs, multiple factors, especially the small molecule FH1, were added in the differentiation medium during hepatic specification and maturation (**Figure 1A**). The matured hiPSC-HLCs presented typical cuboidal hepatocyte morphology and distinctive round small nuclei (**Figure 1A**, lower right). The expression of hepatic markers was determined by different methods including quantitative reverse transcription polymerase chain reaction (qRT-PCR), FACS, immunofluorescence (IF) staining, and enzyme-linked immunosorbent assay (ELISA). The qRT-PCR detected an increasing mRNA levels of human hepatic genes during the hepatic specification and maturation procedure, including albumin (hALB), alpha-1-antitrypsin (hAAT), cytokeratin 18 (hCK18), fumarate dehydrogenase (hFAH), hHNF1α, hHNF4α, asialoglycoprotein receptor (hASGPR), hRXR and sodium taurocholate co-transporting polypeptide (hNTCP) (**Figure 1B**). hRXR and hHNF4α are known to be essential for HBV infection and replication. hNTCP is known to be a receptor that is important for HBV binding and entry (Yan et al., 2012; Xia et al., 2017). Over 90% of cells expressed both hALB and hNTCP as well as hRXR and hHNF4α immediately following maturation, as determined by IF staining and FACS (**Figures 1C–E**). Moreover, immediately after maturation, hiPSC-HLCs showed a remarkable capability of secreting the plasma proteins, hALB and hAAT, at levels of 0.52 ± 0.07 and 2.01 ± 0.13 pg/cell/day, respectively, as measured by ELISA, which indicated a mature hepatic differentiation (**Figure 1F**). Therefore, the hiPSC-HLCs generated in this study were well-differentiated not only morphologically but also functionally.

### Hepatic Properties Were Maintained in hiPSC-HLCs to Support a Long-Term HBV Infection *in Vitro*

**FIGURE 2:**
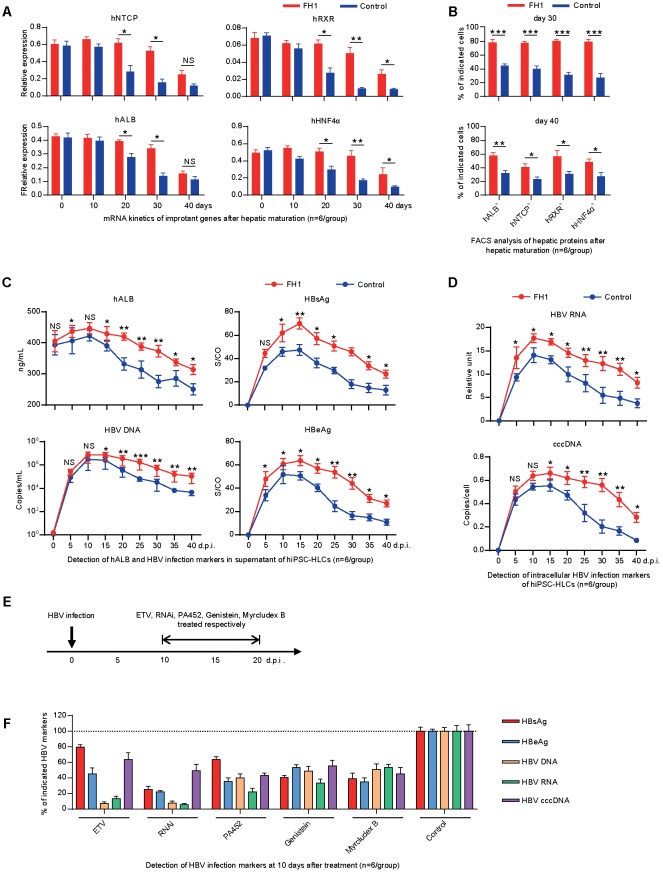
Differentiated hepatic cells were long maintained to support a long-term genotype C HBV infection. **(A)** Fold changes of the mRNA levels of the human hepatic specific genes in hiPSC-HLCs treated with FH1 as compared to that untreated after being normalized to GAPDH (*n* = 6). The primers were as shown in Supplementary Table 2. **(B)** FACS analysis for the positive ratio of the hepatic specific genes in hiPSC-HLCs treated with FH1 as compared to that untreated (*n* = 6). **(C)** Protein levels of hALB, HBV DNA, HBsAg, and HBeAg in the supernatant, and **(D)** intracellular HBV RNA and cccDNA of HBV-infected hiPSC-HLCs at different time points (MOI = 200, *n* = 6). **(E)** Schematic design of the *in vitro* ETV, RNAi, PA452, genistein, and Myrcludex B treatments from days 10 to 20 after HBV infection. **(F)** Detection of relative HBV infection marker suppression of the indicated antivirals at 10 days after treatment (*n* = 6). S/CO, signal-to-cutoff. ^∗^*P* < 0.05, ^∗∗^*P* < 0.01, ^∗∗∗^*P* < 0.001.

Previous studies have shown that PHHs can only support HBV infection *in vitro* for 5–10 days due to the rapid loss of hepatocyte-specific functionality after they are isolated from their *in vivo* microenvironment and adapted to cell culture conditions (Gripon et al., 1988; Allweiss and Dandri, 2016; Xia et al., 2017). To maintain the differentiated hepatic properties, functional small molecule FH1 was added in the cell culture medium. qRT-PCR and FACS methods were employed to determine the level of hepatic specific factors: hNTCP, hRXR, hHNF4α, and hALB. As shown in **Figure 2A**, the qRT-PCR results showed that hiPSC-HLCs treated with FH1 maintained relatively high mRNA levels of hNTCP, hRXR, hHNF4α, and hALB for over 30 days. FACS results showed that approximately 80% of cells treated with FH1 have expressed the four factors for 30 days after hepatic maturation while less than 50% of control cells were co-positive (**Figure 2B**, upper panel). The FH1 kept more than 50% hiPSC-HLCs to produce the four factors till day 40 (**Figure 2B**, lower panel). These results demonstrated that the functional small molecule FH1 can help hiPSC-HLCs to maintain a relatively mature hepatic differentiation state for about 40 days.

To determine whether the hiPSC-HLCs support a long-term infection of HBV, the cells were cultured with or without FH1 and infected with HBV at MOI of 200 (genome equivalents). The kinetics of hALB, HBV DNA, and HBV protein were measured every 5 days for 40 days. As shown in the upper panel of **Figure 2C**, hiPSC-HLCs cultured with FH1 maintained a higher level of hALB in the supernatant than that in controls. Consistently, hiPSC-HLCs with FH1 yielded higher HBV DNA, surface antigen (HBsAg) and e antigen (HBeAg) than those in controls (**Figure 2C**). As we expected, hiPSC-HLCs with FH1 showed higher yield of intracellular HBV RNA or cccDNA than that in controls (**Figure 2D**). These results indicated that the maintenance of hepatic differentiation state was essential to support a long-term HBV infection *in vitro*.

To know whether the cell hiPSC-HLCs can be used to evaluate anti-HBV effects of the antiviral drugs, we infected the hiPSC-HLCs and different drugs (host-targeting agents PA452 and genistin, HBV entry inhibitor Myrcludex B, RNAi and nucleoside analog ETV) were administered at the 10 days post-infection (d.p.i.) (**Figure 2E**). At the 20 d.p.i., HBV DNA, proteins, and RNA were examined. The levels of the viral DNA, RNA, and proteins from the drug-treated group were compared with that from non-treated group. The five drugs displayed a significant inhibitory effect on HBV replication and antigens expression (**Figure 2F**). These antiviral results showed a similar pattern to a recent *in vitro* study on hiPSC-HLCs (Xia et al., 2017), and further confirmed our hiPSC-HLCs can be used for testing anti-HBV drugs.

### Generation of Chimeric Human Liver in Mice: hHLC-FRGS Mice

**FIGURE 3:**
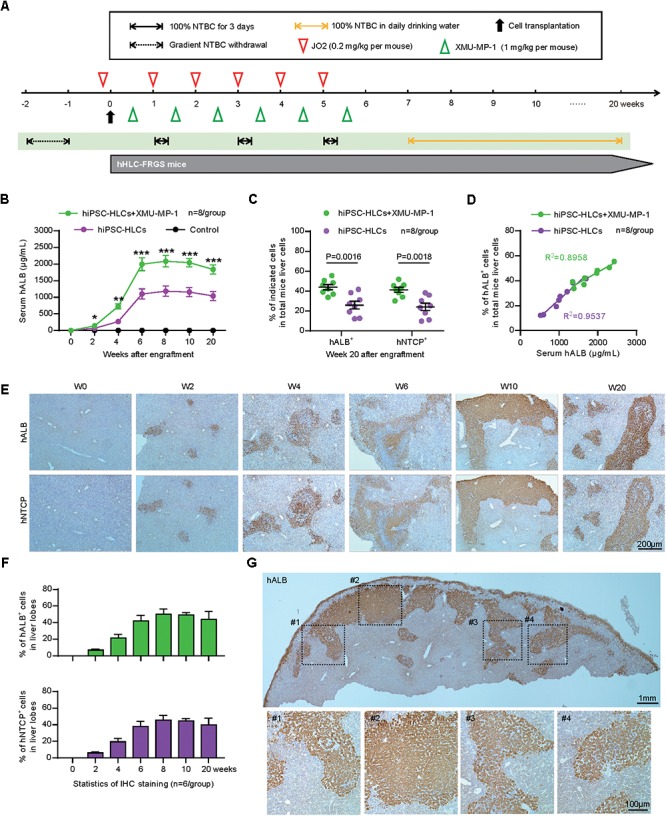
Generation and characterization of human liver chimeric hHLC-FRGS mice. **(A)** Schematic design of hiPSC-HLCs engraftment: NTBC cycled off, JO2 and XMU-MP-1 treatment at the indicated time points. **(B)** Serum hALB levels of hHLC-FRGS mice with or without XMU-MP-1 treatment from week 0 to 20 post-engraftment by ELISA (*n* = 8). **(C)** FACS analysis of the hALB and hNTCP positive ratio of liver cells perfused from hHLC-FRGS mice with or without XMU-MP-1 treatment at week 20 post-engraftment (*n* = 8). **(D)** Linear regression analysis for the relationship between the serum hALB levels and hALB positive ratio of liver cells (*n* = 8). **(E)** IHC staining for hALB and hNTCP positive cells in liver tissues collected from hHLC-FRGS mice with XMU-MP-1 treatment by partial hepatectomy collected by partial hepatectomy at indicated time points from week 0 to 20 post-engraftment (bar = 200 μm), and **(F)** statistics for the different liver lobes (*n* = 6). **(G)** Overall and regional images of IHC staining for hALB positive cells in whole liver lobe (bar = 1 mm or 100 μm). ^∗^*P* < 0.05, ^∗∗^*P* < 0.01, ^∗∗∗^*P* < 0.001.

To generate a chimeric human liver in a mouse model for *in vivo* studies of HBV pathogenesis, hiPSC-HLCs were engrafted into FRGS mice by splenic injection. Liver injury was induced by NTBC cycling and mouse CD95 antibody JO2 to kill part of mouse liver cells in order to provide space for the implanted hiPSC-HLCs to expand. To enhance the expansion of the implanted hiPSC-HLCs, the engrafted mice were given small molecule XMU-MP-1 weekly, which was demonstrated to be capable of augmenting hepatocyte expansion in our previous studies (Fan et al., 2016; **Figure 3A**). Over 90% of hiPSC-HLCs engrafted mice with or without XMU-MP-1 have survived for over 20 weeks with normal body weight and liver function (Supplementary Figure 1). A tumorigenicity assay showed that no tumorigenesis was detected in main organs (Supplementary Figure 2). The serum hALB concentration at week 6 post-engraftment was 1997.2 ± 195.6 mg/mL in the hHLC-FRGS mice treated with XMU-MP-1 and maintained at 1839.8 ± 140.9 mg/mL or above until week 20, whereas the serum concentration of hALB in mice without XMU-MP-1 treatment is 1099.6 ± 154.6 mg/mL at week 6 and maintained at a level of 1041.4 ± 136.2 mg/mL or less till week 20 (**Figure 3B**). The differences of the hALB concentrations in serum between the XMU-MP-1 treated and that not treated were statistically significant.

Then we further investigate the percentile of the hiPSC-HLCs in the chimeric liver producing hALB and hNTCP. Herein, we perfused the liver of hHLC-FRGS mice at week 20 after engraftment and isolated liver cells to examine hALB and hNTCP by FACS and qRT-PCR. The FACS results showed that 44.1 ± 2.6% of liver cells from the XMU-MP-1 treated mice were positive for hALB, whereas only 25.9 ± 3.9% positive ones in the untreated mice (**Figure 3C**). The chimerism of hNTCP positive cells in XMU-MP-1 treated and untreated mice showed similar levels (**Figure 3C**). A linear correlation between the serum hALB levels and ratio of hALB positive cells in liver was found in both the untreated and XMU-MP-1 treated hHLC-FRGS mice (**Figure 3D**). In addition, the hALB positive cells collected from the hHLC-FRGS mice also expressed important human hepatic genes include hAAT, hNTCP, hHNF4α, and hRXR, which expressions had maintained for at least 20 weeks (Supplementary Figure 3).

Livers of hHLC-FRGS mice treated with XMU-MP-1 were collected from week 0 to 20 after engraftment. Immunohistochemistry (IHC) assays were performed to examine hALB and hNTCP, and the results exhibited a robust expansion of implanted hiPSC-HLCs from week 0 to 6 (**Figure 3E**). The positive cells were maintained at 41.9 ± 6.8% until week 20 post-engraftment (**Figure 3E**). Statistical analyses of different views in the lobes and the whole lobe further confirmed the stable chimerism of implanted hiPSC-HLCs (**Figures 3F,G**). Thus, we generated human liver chimeric hHLC-FRGS mice with approximately 40% of hiPSC-HLCs chimerism within 6 weeks. The implanted hiPSC-HLCs maintained a relatively mature hepatic differentiation state and considerable chimerism.

### hHLC-FRGS Mice Support the Chronic HBV Infection

**FIGURE 4:**
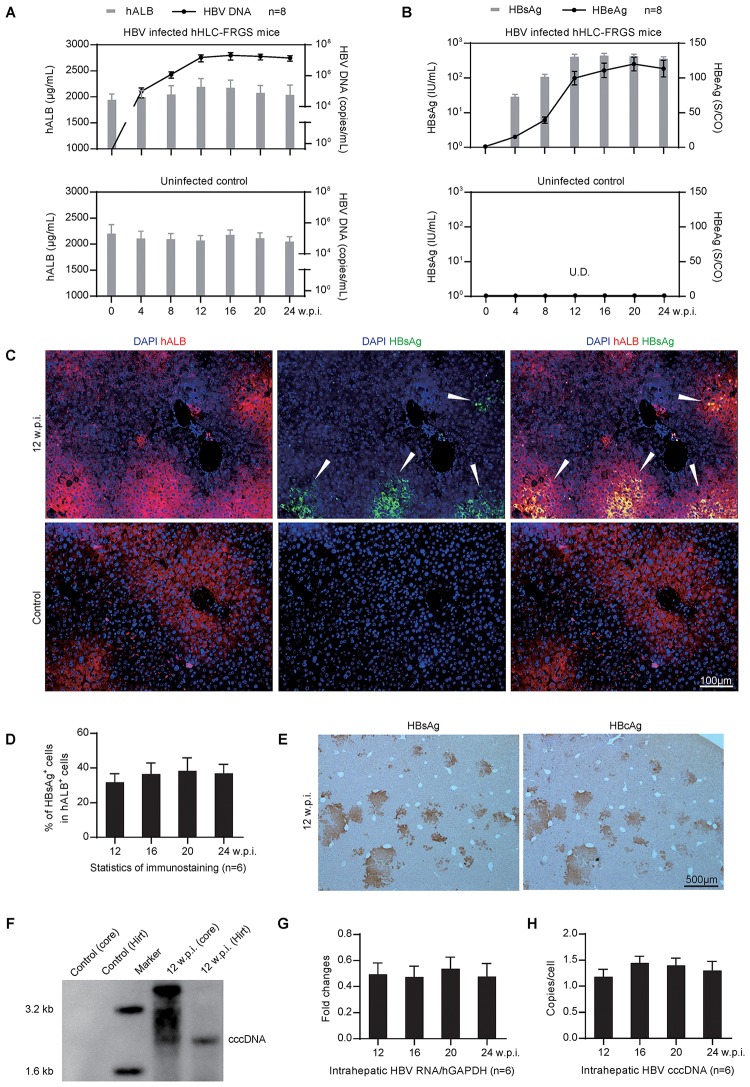
Genotype C HBV infection in hHLC-FRGS mice. **(A)** Serum hALB and HBV DNA from the HBV infected (upper) or the uninfected (lower) hHLC-FRGS. **(B)** HBsAg and HBeAg levels from the HBV infected (upper) or the uninfected (lower) hHLC-FRGS by ELISA and qRT-PCR from 0 to 24 w.p.i. (*n* = 8). **(C)** ICC for hALB (red) and HBsAg (green) expression in frozen sections of liver tissues collected from uninfected control (lower) and HBV-infected (upper) hHLC-FRGS mice at 12 w.p.i. (bar = 100 μm), and **(D)** statistics for the different liver lobes collected by partial hepatectomy at the indicated time points (*n* = 6). **(E)** IHC staining for HBsAg (left) and HBcAg (right) expression in serial sections of liver tissues collected from HBV-infected hHLC-FRGS mice at 12 w.p.i. (bar = 500 μm). **(F)** Southern blot analysis of hALB positive cells collected from perfused liver cells of uninfected control and HBV-infected hHLC-FRGS mice at 12 w.p.i., HBV total DNA (core DNA) and cccDNA (by Hirt method) were isolated for detection with 3 × 10^6^ cells for each lane. **(G,H)** Quantitative analysis of the HBV RNA and cccDNA levels in hALB positive cells collected from HBV-infected hHLC-FRGS mice from 12 to 24 w.p.i. (*n* = 6). The primers were as shown in Supplementary Table 2.

Next, we need to determine whether the hHLC-FRGS mice can be infected by HBV. To that end, hHLC-FRGS mice were intraperitoneally inoculated with the purified HBV viron (genotype C). The HBV DNA, HBsAg, and HBeAg were examined as shown in **Figures 4A,B**. The infected hHLC-FRGS mice with approximately 2000 μg/mL serum hALB (**Figure 4A**, upper panel, gray bar) and were productively amplifying HBV infection markers for a 24-week course. Serum HBV DNA levels increased to 10^6^–10^8^ copies/mL (**Figure 4A**, upper panel, black line), serum HBsAg levels increased to 10^2^–10^3^ IU/mL (**Figure 4B**, upper panel, gray bar), and HBeAg levels increased to 100–150 signal-to-cutoff (S/CO; **Figure 4B**, upper panel, black line) at week 16; these levels of HBV production were maintained for 24 w.p.i. As a control for the specificities of HBV DNA and HBV proteins, the uninfected hHLC-FRGS mice with similar serum hALB levels were used (**Figures 4A,B**, lower panel). These results from the serological viral analyses demonstrated that HBV infected the hHLC-FRGS mice and the established a chronic infection in hHLC-FRGS.

To visualize the HBV infection in the chimeric liver, we performed IHC after the liver tissues collected from the HBV-infected hHLC-FRGS mice at 12 w.p.i. to show human liver protein, hALB (red) and HBV protein, HBsAg (green). As shown by the arrowheads in **Figure 4C**, HBsAg were detected in the hALB positive cells. After counting the hALB positive and hALB/HBsAg duel positive cells, we found that 30–40% of hALB positive cells in liver lobes were positive for HBsAg; this ratio was maintained until 24 w.p.i. (**Figure 4D**). The IHC staining of serial sections showed that over 90% of HBsAg positive cells were also positive for HBcAg at 12 w.p.i. (**Figure 4E**).

It is important to know whether the HBV DNA, RNA, and the cccDNA were generated after infection in the chimeric liver of the hHLC-FRGS, a two-step liver perfusion (Azuma et al., 2007) was performed and the hALB positive cells were collected by FACS. A Southern blot assay was conducted to examine the total HBV DNA and cccDNA that are positively shown in **Figure 4F**. The quantitative measurement of hALB positive cells collected at different times post-infection showed that HBV-infected hHLC-FRGS mice maintained stable intrahepatic HBV cccDNA and RNA levels from 12 to 24 w.p.i. (**Figures 4G,H**). In addition, we also tested the infections of genotypes A, B, and D HBV in hHLC-FRGS mice, we found that the mice also supported their chronic infection, showing similar viremia trends in a 24-week course (Supplementary Figure 4). Therefore, the hHLC-FRGS mouse model was demonstrated capable to establish and support a chronic HBV infection.

### Preventing Viral Spreading in Initial HBV Infection

**FIGURE 5:**
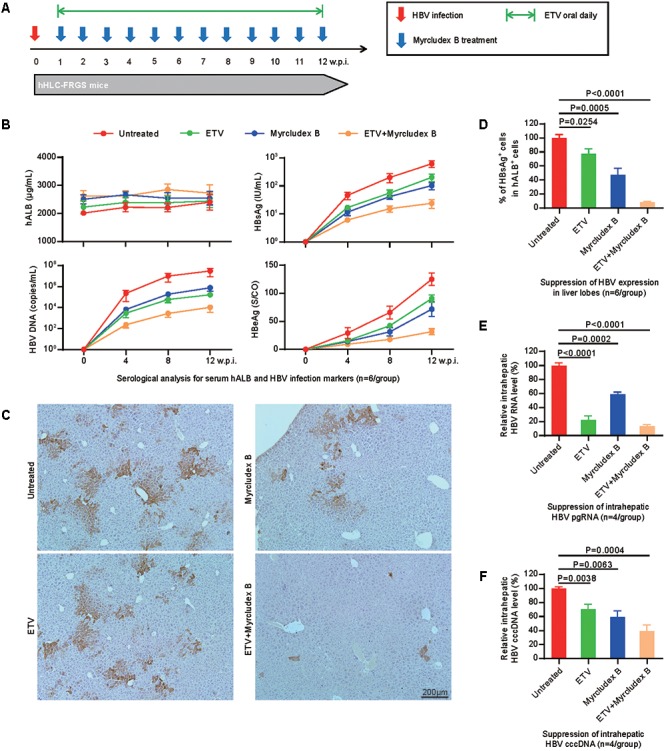
Effects of anti-HBV drugs tested in the hHLC-FRGS. **(A)** Schematic design of ETV and/or Myrcludex B treatment in hHLC-FRGS mice from 1 to 12 w.p.i. Myrcludex B was injected weekly, and ETV was added daily in drinking water. There are four groups of HBV-infected hHLC-FRGS mice: untreated, Myrcludex B single treated, ETV single treated, and combined Myrcludex B and ETV treated. **(B)** Serum hALB, HBV DNA, HBsAg, and HBeAg levels of HBV-infected hHLC-FRGS mice with or without treatment were measured by ELISA and qRT-PCR from 0 to 12 w.p.i. (*n* = 6). **(C)** IHC staining for HBsAg expression in sections of liver tissues collected from HBV-infected hHLC-FRGS mice with or without treatment at 12 w.p.i. (bar = 200 μm), and **(D)** statistics for different liver lobes collected by partial hepatectomy (*n* = 6). **(E)** Quantitative analysis of HBV RNA and **(F)** cccDNA levels in hALB positive cells collected from HBV-infected hHLC-FRGS mice with or without treatment at 12 w.p.i. (*n* = 6).

Subsequently, we asked whether the hHLC-FRGS mice can be used to evaluate the effect of antiviral drugs on HBV. To know that, we chose an well demonstrated HBV entry inhibitor, Myrcludex B that prevents viral spreading at the early time of infection and the clinical viral replication inhibitor ETV, which have been tested in the hiPSC-HLCs to be effective as shown in **Figure 2F**. The mice were infected with HBV, 1 week later the drugs were administered to the mice (Myrcludex weekly and/or ETV daily) as shown in **Figure 5A**. Untreated HBV-infected hHLC-FRGS mice with similar serum hALB levels were used as controls (**Figure 5B**). As shown in **Figure 5B**, serum hALB levels maintained stable, the productions of HBV DNA and proteins were significantly reduced. The results of combination of Myrcludex B and ETV treatment showed that the two drugs may be synergistic in anti-HBV (**Figures 5B,C**). In contrast to the untreated mice, IHC staining of fixed liver tissues collected at 12 w.p.i. showed that ETV, Myrcludex B, and the combined treatment respectively decreased 22.4 ± 7.1, 52.8 ± 9.2, and 91.8 ± 1.2% of HBsAg positive cells in liver lobes (**Figures 5C,D**). The suppressive effects of the drugs were also shown by the reduced HBV RNA productions (**Figure 5E**) and decreased HBV cccDNA levels: (1) the combined treatment decreased 86.9 ± 2.6% of intrahepatic HBV RNA, Myrcludex B decreased 41.3 ± 3.4%, ETV decreased 77.9 ± 5.9% of intrahepatic HBV RNA (**Figure 5E**); and (2) the combined treatment decreased 60.8 ± 8.6% of intrahepatic HBV cccDNA, Myrcludex B decreased 41.1 ± 9.1% and ETV decreased 29.6 ± 7.2% of intrahepatic HBV cccDNA (**Figure 5F**).

It is important to know whether the antiviral drugs have any side effects on the mice. To know that, we performed different assays. First, the body weights were not significantly altered by the drugs as shown in Supplementary Figure 5A. Then, liver function assays were performed to examine the levels of ALT, AST, TBIL, TBA, TP, and PT, no significant variations were detected in the mice treated with the drugs and the ones without drugs (Supplementary Figure 5B). Last, H&E staining showed that the liver tissues of the livers from the drug-treated mice and the non-treated ones were similar (Supplementary Figure 5C). Therefore, these results demonstrated that our hHLC-FRGS mice can be used to evaluate anti-HBV drugs and that Myrcludex B and ETV are effective on suppressing HBV replication.

## Discussion

Hepatitis B virus is a hepatotropic virus that infects large population and causes acute and/or chronic diseases in liver after infection (Dienstag, 2008; Trepo et al., 2014). HBV has been designated as an oncovirus because people chronically infected with HBV have a higher risk of liver cirrhosis or HCC. Therefore, although an effective HBV vaccine has been available since 1982 to prevent infection, HBV still threatens human health because most chronic carriers of HBV have potential to develop cirrhosis and HCC (Liang et al., 2015; Loomba and Liang, 2017).

Difficulties in developing infectious cell lines and animal models have hampered research on HBV. PHHs are the closest to natural statues for HBV infection and were used for studying HBV entry, searching HBV receptors, and testing anti-HBV drugs. However, PHHs are difficult to culture and obtain, and easy to lose hepatic function (Ochiya et al., 1989; Ren and Nassal, 2001; Schulze-Bergkamen et al., 2003; Yan et al., 2012; Konig et al., 2014). Human hepatoma cell lines such as HepG2, Chang, Hep3B, and HuH are easy to culture and were used for HBV production and drug screening (Sells et al., 1988; Chouteau et al., 2001; Sun and Nassal, 2006). Based on the human hepatoma cell lines, several cell lines were generated by stable expressing hNTCP, but they lack the properties of PHHs and are malignant, which limits their applications in HBV research (Nkongolo et al., 2014; Watashi et al., 2014; Xia et al., 2017). To solve the shortcomings of the PHHs and hepatoma cell lines, HLCs derived from human stem cells, especially from hiPSCs have been developed and demonstrated capable to support *in vitro* HBV infection (Paganelli et al., 2013; Shlomai et al., 2014; Sakurai et al., 2017; Xia et al., 2017). In the present study, we differentiate the hiPSCs to hiPSC-HLCs. Then, we performed different experiments including FCAS, qRT-PCR, and IHC, and demonstrated that the hiPSC-HLCs expressed hepatic genes such as hALB and hAAT (**Figure 1B**), critical factors for HBV infection such as hNTCP, hRXR, and hHNF4α (**Figure 1B**), and can support HBV infection for at least 40 days (summarized in **Figures 1**, **2**). Different viral products (viral DNA, cccDNA, RNA, and viral proteins) were detected in hiPSC-HLCs persistently for 40 days post-HBV infection (**Figure 2**).

A small animal model for HBV infection is critical in not only exploring the antiviral medicines but also ascertaining the HBV pathogenesis. The development of HBV animal models has experienced a long and difficult time due to the species specificity of HBV infection. A transgenic mouse model was generated previously and has been useful for investigating HBV pathogenesis and for developing antiviral drugs (Guidotti et al., 1995, 1996). Scientists have improved the transgenic mode by providing syngeneic unprimed splenocytes so that the model was closer to the chronic HBV infection in immunodeficient mice (Larkin et al., 1999), the virus replication were still at a low level and no cccDNA was detected. Then, chimeric mouse models with PHHs were developed (Tsuge et al., 2005; Bissig et al., 2010; Kosaka et al., 2013), these models were useful to study the HBV virology and evaluate anti-HBV drugs (Sugiyama et al., 2007; Tabuchi et al., 2008; Robinet and Baumert, 2010; Lutgehetmann et al., 2011). However, the varied expansion capacity of PHHs from different donors in the mouse liver and limited resource of PHHs are the shortcomings of these models (de Jong et al., 2010; Dandri and Petersen, 2012; Allweiss and Dandri, 2016; Thomas and Liang, 2016).

**FIGURE 6:**
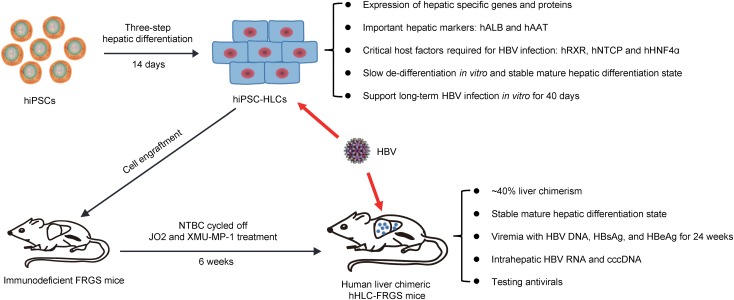
Summary of hiPSC-HLCs and hHLC-FRGS mice supporting HBV infection. hiPSCs were induced to differentiate to hepatocyte-like cells (HLCs). The hHLCs are characterized by examining their expression of hALB, hAAT, hRXR, hNTCP, and hHNF4α. This study included the generation and characterization of hiPSC-HLCs, the maintenance of long-term hepatic differentiation state and *in vitro* HBV infection, generation of human liver chimeric hHLC-FRGS mice by hiPSC-HLCs engraftment, establishment of chronic HBV infection.

Human stem cell-derived hepatocyte-like cells have been demonstrated to be expandable in the mouse liver and permissive for *in vivo* HCV infection (Basma et al., 2009; Carpentier et al., 2014; Yan et al., 2017). However, these previous hHLCs engrafted mice were not applied to HBV infection or therapy. In our present research, we successfully established a human chimeric mouse, hHLC-FRGS, by implanting the hiPSC-HLCs to mouse liver for HBV infection. First, the HLCs were highly expandable after engraftment when weekly treated with the functional small molecule XMU-MP-1 (**Figure 3**). In our chimeric mouse model, the percentage of the hHLCs in the chimeric liver reached about 40% within 6 weeks (**Figure 3F**). Furthermore, the hHLCs survive in the mouse liver for a long time (over 20 weeks) and showed stable human liver functions and no evidence of tumorigenesis in the main organs (**Figure 3F** and Supplementary Figure 2), implying that it can be used for chronic HBV infection. In Addition, our *in vitro* and *in vivo* studies showed that the functional small molecule FH1 or mouse liver environment can help maintain the mature hepatic differentiation state of hiPSC-HLCs (summarized in **Figures 1**–**3**), which is considered to be a critical precondition to support long-term HBV infection. The development and characteristics of the hiPSC-HLCs and the hHLC-FRGS mice system supporting HBV infection are summarized in **Figure 6**.

As expected, the hHLC-FRGS mouse permitted HBV infection in the chimeric liver. The viral production was detected by multiple methods: FACS, IHC, qRT-PCR, and Southern blot assays (**Figure 4**). Due to the persistence of HBV infection for over 20 weeks, we conclude that this humanized mouse model is a suitable model of chronic HBV infection. Importantly, not only were the HBV DNA, antigens and RNA increased post-HBV infection in the mouse, but also the cccDNA was detected. As HBV cccDNA has been demonstrated the key factor to establish sustained HBV infection and important target of antiviral studies (Cai et al., 2016; Qi et al., 2016; Guo et al., 2017), our present results suggest that hiPSC-HLCs and hHLC-FRGS mouse will be useful infectious system to monitor the effects of drugs on HBV infection. Moreover, the prevention of viral spreading is one of the most important approaches for suppressing HBV infection (Durantel and Zoulim, 2016). In this study, the combination of the well-demonstrated HBV entry inhibitor Myrcludex B with the clinical drug ETV showed efficient blockage of HBV spread in hHLC-FRGS mice (**Figure 5**).

## Conclusion

Our *in vitro* and *in vivo* experiments demonstrated that hiPSC-HLCs and hHLC-FRGS mice support productive HBV infections, mimic a chronic HBV-caused viremia, and can be used to evaluate the effects of anti-HBV drugs. Due to its persistent mature hepatic differentiation and high rate of hHLCs in the chimeric liver, hHLC-FRGS mice may be used for studies of other hepatotropic virus infections.

## Author Contributions

LY and XuL performed the cell culture system and measurement of hepatocyte markers. LY, XuL, LZ, YZ, and KW performed the animal studies. LZ, XiL, YC, and JC performed the HBV infection and measurement of HBV infection markers. JZ, WH, and HZ performed the statistical analysis. YZ, QT, and TC wrote the manuscript. QY, QT, TC, and NX supervised the project.

## Conflict of Interest Statement

The authors declare that the research was conducted in the absence of any commercial or financial relationships that could be construed as a potential conflict of interest.
