# Supplementary material for: A Chimeric Humanized Mouse Model by Engrafting the Human Induced Pluripotent Stem Cell-Derived Hepatocyte-Like Cell for the Chronic Hepatitis B Virus Infection
Source: Front Microbiol. 2018 May 8;9:908. doi: 10.3389/fmicb.2018.00908 (PMC5952038; doi:10.3389/fmicb.2018.00908)
Supplement: Supplementary file 1 [file Presentation_1.PDF]

Supplementary Materials

A Chimeric Humanized Mouse Model by Engrafting the Human Induced Pluripotent Stem Cell-derived Hepatocyte-like Cell for the Chronic Hepatitis B Virus Infection

Lunzhi Yuan[1¶], Xuan Liu[1¶], Liang Zhang[1], Xiaoling Li[1], Yali Zhang[1], Kun Wu[1], Yao chen[1], Jiali Cao[1], Wangheng Hou[1], Jun Zhang[1], Hua Zhu[3], Quan Yuan[1*], Qiyi Tang[2*], Tong Cheng[1*], Ningshao Xia[1]

- **Supplementary Materials and Methods**
  - *Detection of hALB, hAAT and other proteins secreted by hiPSC-HLCs*
  - *FACS analysis*
  - *qRT-PCR*
  - *Collection of liver cells by collagenase perfusion*
  - *IF staining*
  - *IHC and H&E staining*
  - *Detection of HBV infection markers*
- **Supplementary Figure Legends**
- **Reference for Supplementary Materials**
- **Supplementary Tables**
  - *Supplementary Table.1. Antibodies.*
  - *Supplementary Table.2. Primers for qRT-PCR.*
- **Supplementary Figures**

Supplementary Materials and Methods

Detection of hALB, hAAT and liver function markers

The levels of hALB and hAAT in mice serum and cell culture supernatant were measured by ELISA Kits from Bethyl. The levels of hAFP, ALT, AST, TBIL and TBA in mice serum were measured by Kits from Wantai, China.

FACS analysis

The cells for FACS analysis were incubated at 4℃ for 30 minutes with indicated antibodies. They were then rinsed with PBS twice and analyzed with a FACS instrument (Facsaria III, BD). The details of indicated antibodies were showed in Supplementary Table.1.

qRT-PCR

Total RNA was extracted from tissues or purified cells with TRIzol reagent (Invitrogen) according to the manufacturer's instructions and used for cDNA synthesis with a SuperScript First-Strand Synthesis System (Invitrogen) as described. qRT-PCR was performed on a 7500 Fast Real-Time PCR system. The details of indicated primers were showed in Supplementary Table.2.

Collection of liver cells by collagenase perfusion

Cells from repopulated primary recipients were harvested with a standard collagenase perfusion protocol [1]. Briefly, the liver was perfused with calcium- and magnesium-free Earle’s balanced salt solution (EBSS) supplemented with 0.5 mM EGTA and 10 mM HEPES for 5 min. The solution was changed to EBSS supplemented with 0.1 mg/ml collagenase IV (Sigma-Aldrich) and 0.05 mg/mL DNase I (Sigma-Aldrich) for 10 min. The liver was gently minced in the second solution and filtered through 70 mm and 40 mm nylon mesh sequentially. After 150g centrifugation for 5 min, the pellet was washed twice at 50g for 2 min. The number and viability of cells were assessed by Trypan blue exclusion test.

IF staining

Cells cultured on slides were fixed by 4% paraformaldehyde for 20 minutes, incubated with 0.1% Triton-X100 for 10 minutes, incubated with 20% BSA for 30 minutes, and then incubated with the first and second antibodies. Cell nucleus were stained by DAPI

for 3 minutes. Among each step, the slides were washed with PBS for 3 times. For frozen sections, use livers were frozen in OCT (Sakura) and 10-μm cryostat sections were fixed in 4% paraformaldehyde for 15 min at 20°C (used throughout). Sections were permeabilized in 0.1% Triton X-100 for 10 min, then incubated in 0.3% H2O2 for 5 min, then for 20 min in10% goat serum, and then incubated with antibodies. Nuclei was stained by DAPI. The slides were washed by PBS for three times between each two steps. Photomicrographs were taken with an Axioimager microscope (Zeiss) and BX51 microscope (Olympus).

IHC and H&E staining

For paraffin sections, mice tissues were fixed in 4% formaldehyde (PH 7.4) for at least 48 hours. Sections (4 μm) were applied to poly-L-lysine-coated slides. After the sections were dewaxed, rehydrated and washed, endogenous peroxidases were inactivated with 3% H_2O_2 for 10 minutes at room temperature. The sections were then incubated overnight with primary antibodies. The sections were subsequently washed with PBS three times and treated with reagents from an UltraSensitive™ SP Kit (Fuzhou Maixin Biotech, China). After reaction with the DAB chromogen, the sections were rinsed with distilled water, counterstained with haematoxylin. Brown staining indicated positive expression. For H&E staining were predicted by a Kit from Fuzhou Maixin Biotech. Photomicrographs were taken with an Axioimager microscope (Zeiss) and BX51 microscope (Olympus).

Detection of HBV infection markers

HBsAg and HBeAg in mice serum and cell culture supernatant were measured by ELISA Kits from Wantai. HBV DNA in mice serum and cell culture supernatant were measured using a qRT-PCR assay from Premix Ex Taq™ (Takara) as previous described [2]. The primer sequences were 5'-GTT CAA GCC TCC AAG CTG TG-3' and 5'-TCA GAA GGC AAA AAA GAG AGT AAC TC-3'. The probe sequence was 5'-Hex- CCT TGG GTG GCT TTG GGG CAT GGA-BHQ-1-3'. Southern blot performed according to the methods of a previous study using DIG-labelled DNA fragments from the X gene as a probe [3]. Quantification of HBV RNA and cccDNA were produced as previous described [4,5].

Supplementary Figure Legends

Fig. S1. Survival and liver function analysis of hHLC-FRGS mice. (A) Survival analysis of hHLC-FRGS mice with or without XMU-MP-1 treatment from day 0 to 140 after engraftment (n=30/group), FRGS mice without engraftment were set as control (n=30/group), and **(B)** monitor of mice body weight during this period (n=8/group). **(C)** Measurement of six typical liver function markers in serum of long-term survived control and hHLC-FRGS mice (n=8/group). The indicated liver function markers include alanine aminotransferase (ALT), aspartate aminotransferase (AST), total bilirubin (TBIL), bile acid (TBA), total protein (TP) and prothrombin time (PT).

Fig. S2. Investigate the long-term tumourigenicity of hHLC-FRGS mice. (A) Measurement of serum hepatocellular carcinoma marker hAFP and hGPC-3 in serum of hHLC-FRGS mice and control FRGS mice from day 0 to 140 after engraftment (n=8/group). **(B)** Liver tissues were collected by partial hepatectomy from hHLC-FRGS mice and control FRGS mice at day 0, 70 and 140 after engraftment, and measured for mRNA levels of human hepatocellular carcinoma related genes and hGAPDH by qRT-PCR (n=4/group). **(C)** H&E sating of liver tissue collected from hHLC-FRGS mice at day 0 and 140 after engraftment (bar=200μm). **(D)** H&E sating of tissues collected from other main organs include heart, spleen, lung, kidney and colon of hHLC-FRGS mice at day 0 and 140 after engraftment (bar=100μm).

Fig. S3. Hepatic differentiation state of the implanted hiPSC-HLCs. (A) hALB and hAAT synthesis capacity of hiPSC-HLCs right after maturation and collected from hHLC-FRGS mice at day 140 after engraftment were measured by ELISA (n=4/group). **(B)** Relative mRNA levels of human hepatic genes include hALB, hNTCP, hHNF4α and hRXR, and hGAPDH of these two cells were measured by qRT-PCR (n=4/group). There was no significant difference between hiPSC-HLCs right after maturation and collected from hHLC-FRGS mice at day 140 after engraftment.

Fig. S4. Chronic infection of HBV genotype A, B and D were established in hHLC-FRGS mice. (A) Serum hALB, HBV DNA, HBsAg and HBeAg levels of uninfected control and HBV-infected hHLCs-FRGS mice were measured by ELISA and qRT-PCR from 0 to 24 w.p.i. (n=4/group). **(B)** IHC staining for HBsAg expression in serial sections of liver tissues collected from HBV-infected hHLCs-FRGS mice at 24 w.p.i. (bar=200μm), and **(C)** statistics for different liver lobes collected by partial hepatectomy from 12 to 24 w.p.i. (n=6/group).

Fig. S5. Liver physiology of HBV-infected hHLC-FRGS mice with RTV and Myrcludex B treatment. (A) Monitor of body weight of hHLC-FRGS mice from 0 to 12 w.p.i. (n=6/group). **(B)** Measurement of six typical liver function markers in serum of control and hHLC-FRGS mice from 0 to 12 w.p.i. (n=6/group). The indicated liver function markers include ALT, AST, TBIL, TBA, TP and PT. **(C)** H&E sating of liver tissue collected from control and treated hHLC-FRGS mice at week 12 w.p.i. (bar=200μm). There was no significant difference between the untreated control and the three treatment groups.

Reference for Supplementary Materials

1. Azuma H, Paulk N, Ranade A, Dorrell C, Al-Dhalimy M, et al. (2007) Robust expansion of human hepatocytes in Fah-/-/Rag2-/-/Il2rg-/- mice. Nat Biotechnol 25: 903-910.
2. Zhang TY, Yuan Q, Zhao JH, Zhang YL, Yuan LZ, et al. (2016) Prolonged suppression of HBV in mice by a novel antibody that targets a unique epitope on hepatitis B surface antigen. Gut 65: 658-671.
3. Huang CH, Yuan Q, Chen PJ, Zhang YL, Chen CR, et al. (2012) Influence of mutations in hepatitis B virus surface protein on viral antigenicity and phenotype in occult HBV strains from blood donors. J Hepatol 57: 720-729.
4. Wang J, Shen T, Huang X, Kumar GR, Chen X, et al. (2016) Serum hepatitis B virus RNA is encapsidated pregenome RNA that may be associated with persistence of viral infection and rebound. J Hepatol 65: 700-710.
5. Singh M, Dicaire A, Wakil AE, Luscombe C, Sacks SL (2004) Quantitation of hepatitis B virus (HBV) covalently closed circular DNA (cccDNA) in the liver of HBV-infected patients by LightCycler real-time PCR. J Virol Methods 118: 159-167.

Supplementary Table.1

Antibodies	Application	Cat. No.	Source
Purified NA/LE Hamster Anti-Mouse CD95 Clone JO2	Injection	554254	BD
anti-human albumin	IHC, FACS, IF	Ab10241	Abcam
anti-human NTCP	IHC, FACS, IF	HPA042727	Sigma
anti-human HNF4α	FACS, IF	ab92378	Abcam
anti-human RXR	FACS, IF	sc-553	Santa Cruz
anti-HBsAg	IHC	83H12	Zhang, et al. GUT, 2016
anti-HBcAg	IHC	B058601	DAKO
Anti-Rabbit IgG (whole molecule)–TRITC antibody produced in goat	secondary antibody	T6778-1ML	Sigma
Anti-Mouse IgG (whole molecule)−TRITC antibody produced in goat		T5393-1ML	
Anti-Rabbit IgG (whole molecule)–FITC antibody produced in goat		F9887-1ML	
Anti-Mouse IgG (whole molecule)–FITC antibody produced in goat		F9006-2ML	

Supplementary Table.2

Genes	Primer	
hALB	F	TTTATGCCCCGGAACTCCTTTT
	R	ACAGGCAGGCAGCTTTATCAG
hAAT	F	GCCTATGATGAAGCGTTTAGGC
	R	TTCCAGTAATGGACAGTTTGGGT
hHNF4α	F	AACGGACAGATGTGTGAGTGG
	R	CAGGAGCTTATAGGGCTCAGAC
HNF1α	F	GCCACCTGCTGCCATCCAA
	R	TGCAGCCCGTAGTTTAAAC
hFAH	F	CCTACGGCGTCTTCTCGAC
	R	CTGCAAGAACACTCTCGCCT

hNTCP	F	AAGGACAAGGTGCCCTATAAAGG
	R	ACGATCCCTATGGTGCAAGGA
hCK18	F	TCGCAAATACTGTGGACAATGC
	R	GCAGTCGTGTGATATTGGTGTC
hASGPR	F	ATGACCAAGGAGTATCAAGACCTT
	R	TGAAGTTGCTGAACGTCTCTCT
hRXR	F	GGAGGTGAGGGAGGAGTT
	R	GCATGAGTTAGTCGCAGACAT
hAFP	F	CTTGCACACAAAAAGCCCACT
	R	GGGATGCCTTCTTGCTATCTCAT
hGPC-3	F	CTGCTTCAGTCTGCAAGTATGG
	R	GTGGAGTCAGGCTTGGGTAG
hGAPDH	F	GGAGTCAACGGATTTGGTCGT
	R	CACTTGATTTTGGAGGGATCTCG
