# Supplementary material for: A Chimeric Humanized Mouse Model by Engrafting the Human Induced Pluripotent Stem Cell-Derived Hepatocyte-Like Cell for the Chronic Hepatitis B Virus Infection
Source: Front Microbiol. 2018 May 8;9:908. doi: 10.3389/fmicb.2018.00908 (PMC5952038; doi:10.3389/fmicb.2018.00908)

Supplementary Figure 1

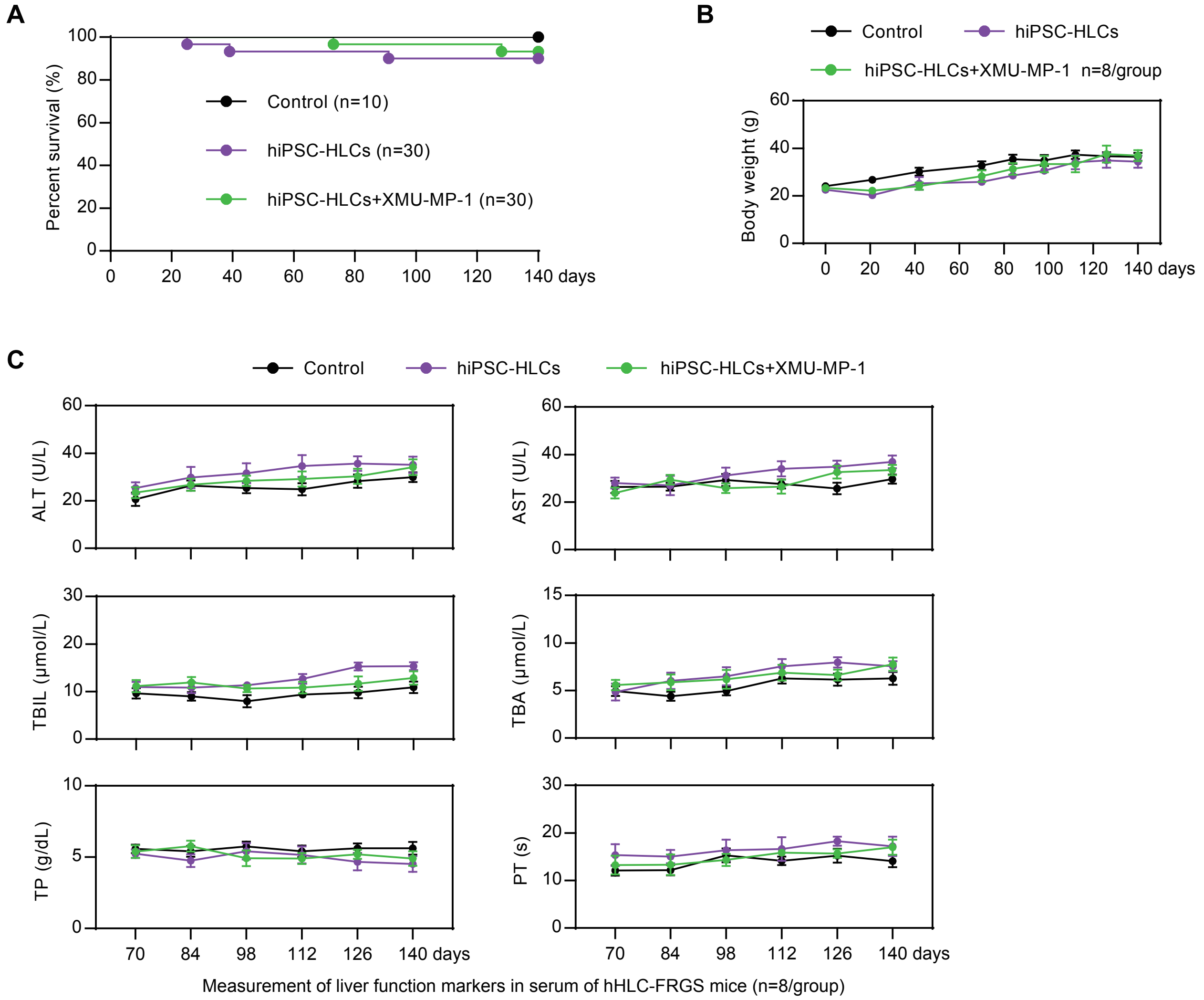

Supplementary Figure 2

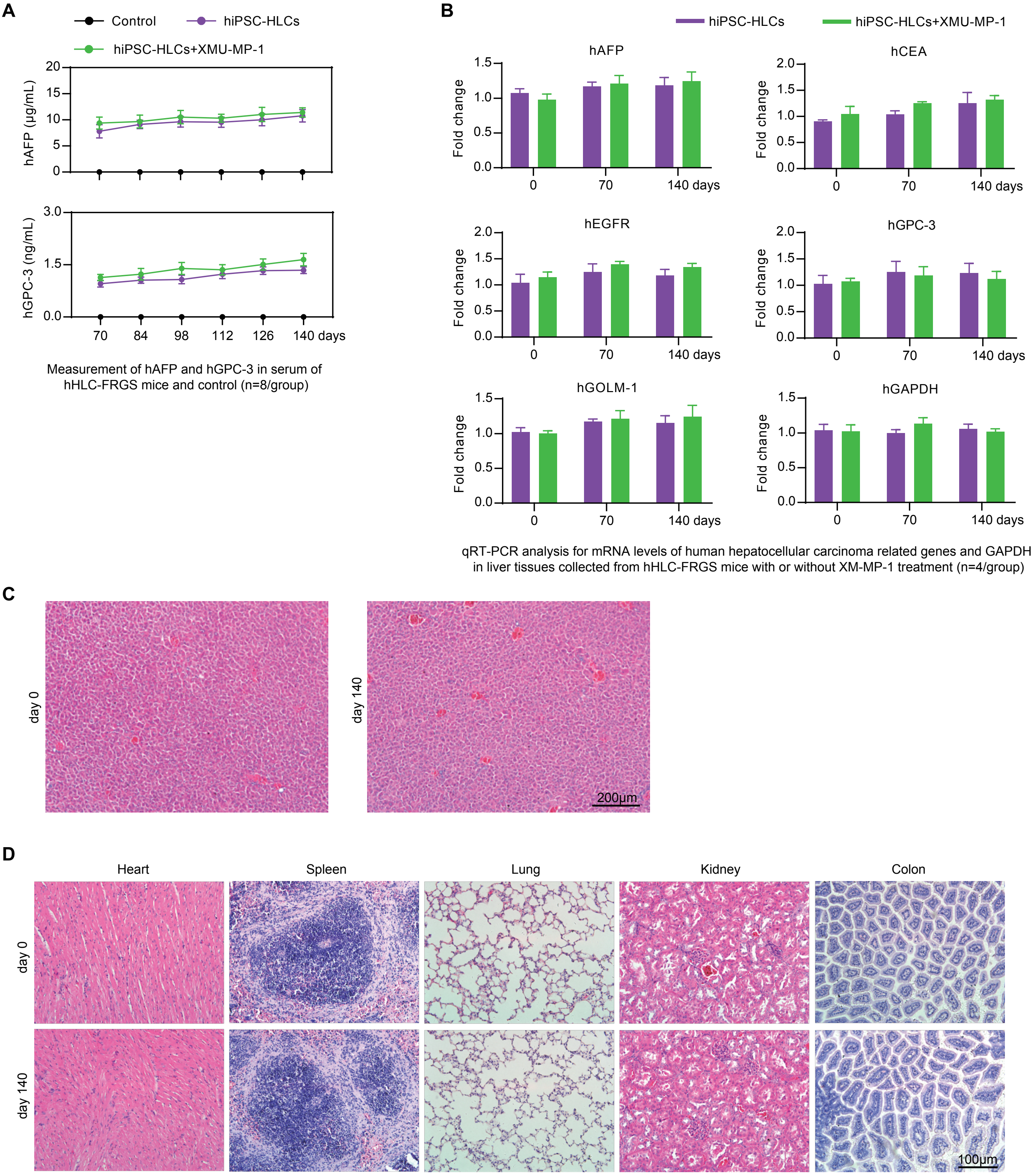

Supplementary Figure 3

A

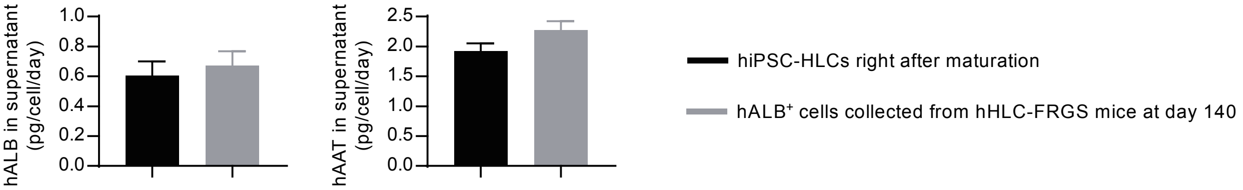

hALB and hAAT synthesis capacity measured by ELISA (n=4/group)

B

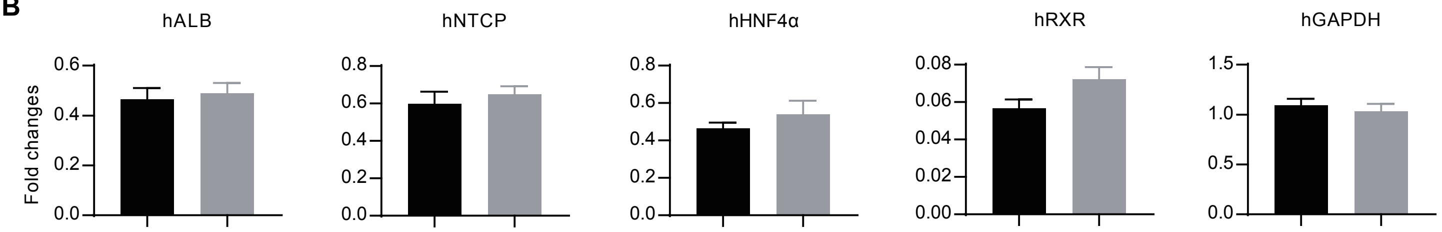

Relative mRNA levels of human hepatic genes and hGAPDH measuredby qRT-PCR (n=4/group)

Supplementary Figure 4

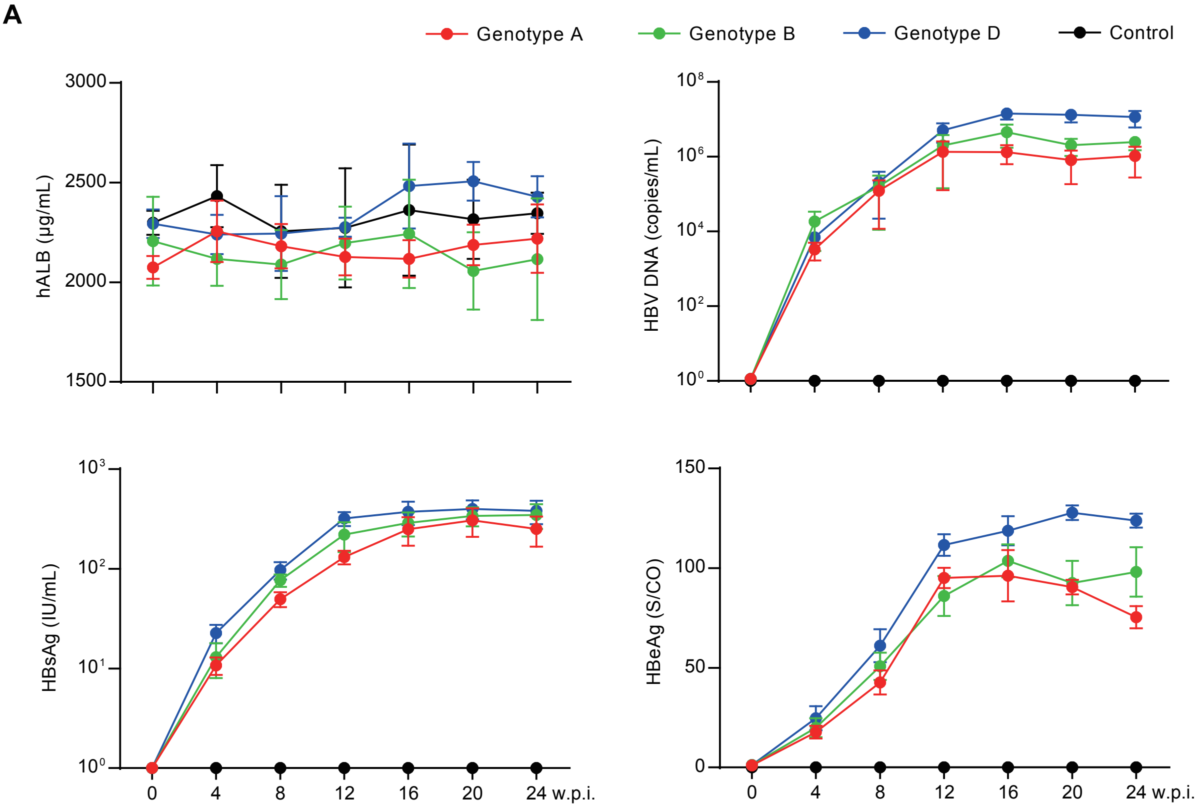

Serum hALB levels and HBV infection markers of control and hHLC-FRGS mice infected with HBV genotype A, B, D (n=4/group)

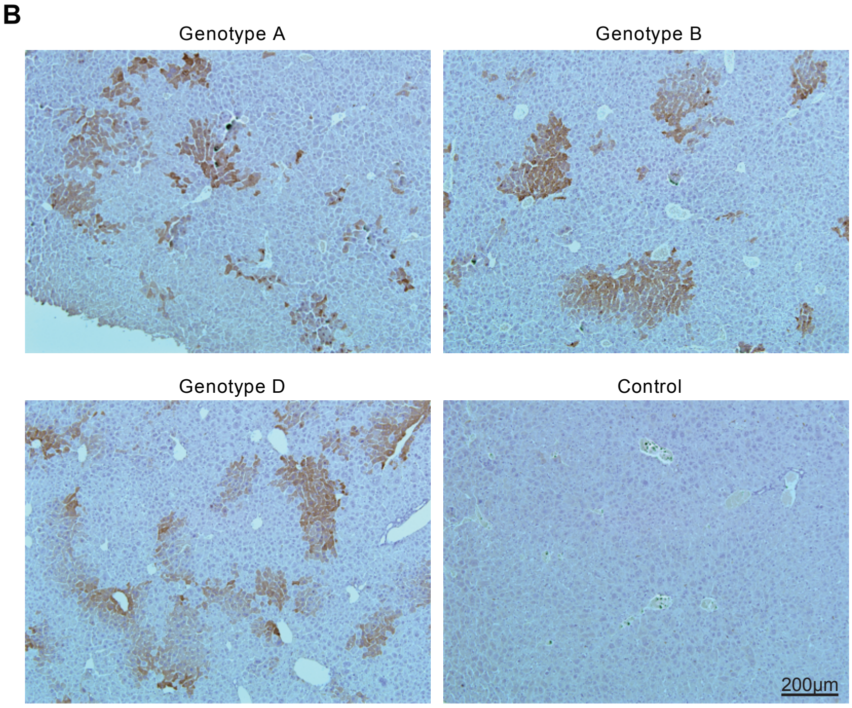

HBsAg expression in liver lobes collected from control and hHLC-FRGS mice infected with HBV genotype A, B, D at 24 w.p,i,

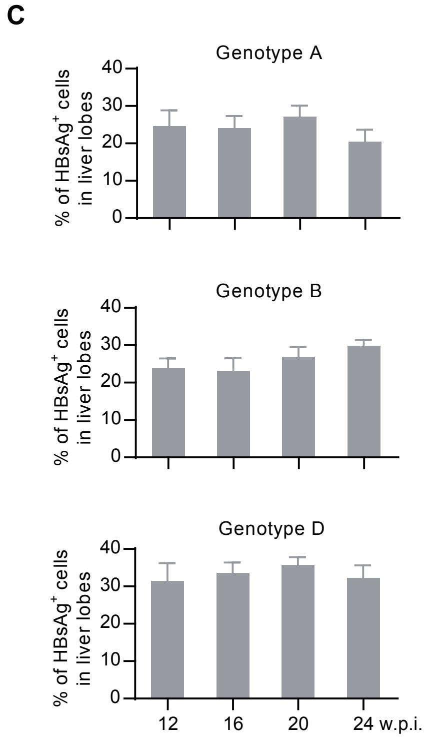

Statistics of immunostaining (n=6/group)

Supplementary Figure 5

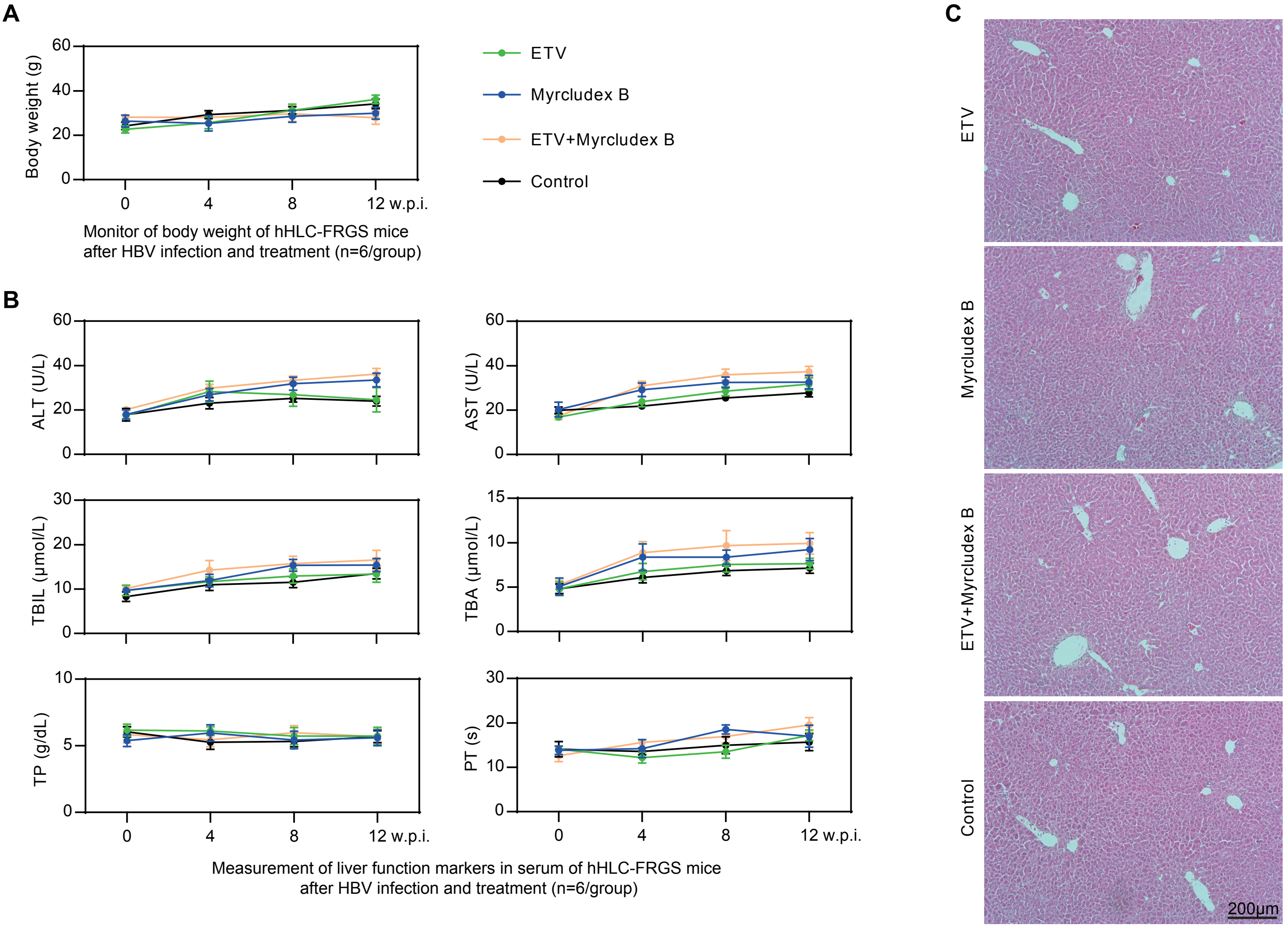

Supplement: Supplementary file 2 [file Image_1.PDF]
